# Piperazinediium dioxamate

**DOI:** 10.1107/S1600536809012513

**Published:** 2009-04-08

**Authors:** S. Murugavel, R. Selvakumar, S. Govindarajan, P. S. Kannan, A. SubbiahPandi

**Affiliations:** a Department of Physics, Thanthai Periyar Government Institute of Technology, Vellore 632 002, India; b Department of Chemistry, Bharathiar University, Coimbatore 641 046, India; c Department of Physics, S.M.K. Fomra Institute of Technology, Thaiyur, Chennai 603 103, India; d Department of Physics, Presidency College (Autonomous), Chennai 600 005, India

## Abstract

The title compound, C_4_H_12_N_2_^2+^·2C_2_H_2_NO_3_^−^, contains a network of doubly protanated piperazinium cations (lying about centres of inversion) and dioxamate anions. The piperazinium dication adopts a typical chair conformation. The crystal structure is stabilized by cation–to–anion N—H⋯O and anion–to–anion N—H⋯O hydrogen bonds.

## Related literature

For related structures, see: Büyükgüngör & Odabaşoğlu (2008 ▶); Wilkinson & Harrison (2007 ▶). For biological applications of piperazines, see: Berkheij *et al.* (2005 ▶); Humle & Cherrier (1999 ▶). For the synthesis of a ligand with two piperazine arms, see: Bharathi *et al.* (2006 ▶). For the use of piperazine derivatives as buffers, see: Good *et al.* (1966 ▶). For the piperazine nucleus and its ability to bind to multiple receptors, see: Dinsmore & Beshore (2002 ▶).
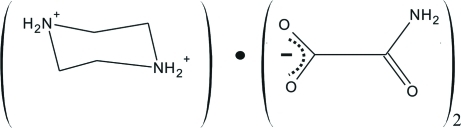


## Experimental

### 

#### Crystal data

C_4_H_12_N_2_^2+^·2C_2_H_2_NO_3_^−^
*M* *_r_* = 264.25
Monoclinic, 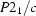

*a* = 6.4323 (4) Å
*b* = 6.7681 (4) Å
*c* = 13.0032 (7) Å
β = 94.488 (2)°
*V* = 564.35 (6) Å^3^
*Z* = 2
Mo *K*α radiation
μ = 0.13 mm^−1^
*T* = 293 K
0.24 × 0.22 × 0.16 mm

#### Data collection

Bruker APEXII CCD diffractometer
Absorption correction: multi-scan (*SADABS*; Sheldrick, 1996 ▶) *T*_min_ = 0.969, *T*_max_ = 0.979
9313 measured reflections
2606 independent reflections
2197 reflections with *I* > 2σ(*I*)
*R*_int_ = 0.021

#### Refinement

*R*[*F* ^2^ > 2σ(*F* ^2^)] = 0.041
*wR*(*F* ^2^) = 0.119
*S* = 1.09
2606 reflections
82 parameters
3 restraints
H-atom parameters constrained
Δρ_max_ = 0.36 e Å^−3^
Δρ_min_ = −0.34 e Å^−3^

### 

Data collection: *APEX2* (Bruker, 2004 ▶); cell refinement: *APEX2* and *SAINT* (Bruker, 2004 ▶); data reduction: *SAINT* and *XPREP* (Bruker, 2004 ▶); program(s) used to solve structure: *SHELXS97* (Sheldrick, 2008 ▶); program(s) used to refine structure: *SHELXL97* (Sheldrick, 2008 ▶); molecular graphics: *ORTEP-3* (Farrugia (1997 ▶) and *PLATON* (Spek, 2009 ▶); software used to prepare material for publication: *SHELXL97* and *PLATON*.

## Supplementary Material

Crystal structure: contains datablocks global, I. DOI: 10.1107/S1600536809012513/lx2097sup1.cif

Structure factors: contains datablocks I. DOI: 10.1107/S1600536809012513/lx2097Isup2.hkl

Additional supplementary materials:  crystallographic information; 3D view; checkCIF report

## Figures and Tables

**Table 1:** Hydrogen-bond geometry (Å, °)

*D*—H⋯*A*	*D*—H	H⋯*A*	*D*⋯*A*	*D*—H⋯*A*
N1—H1*A*⋯O1^i^	0.86	2.24	3.0232 (9)	152
N1—H1*B*⋯O3^ii^	0.86	2.07	2.8622 (8)	153
N2—H2*A*⋯O1^iii^	0.90	2.37	3.0589 (8)	133
N2—H2*A*⋯O2^iii^	0.90	1.94	2.7475 (9)	149
N2—H2*B*⋯O1^iv^	0.90	1.87	2.7509 (9)	164

Symmetry codes: (i) 
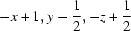
; (ii) 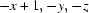; (iii) 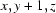; (iv) 
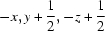
.

**Fig. 1.**
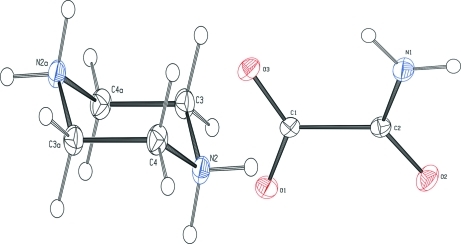
The molecular structure of the title compound with the atom numbering scheme. Displacement ellipsoids are drawn at the 50% probability level. H atoms are presented as a small cycles of arbitrary radius.

**Fig. 2.**
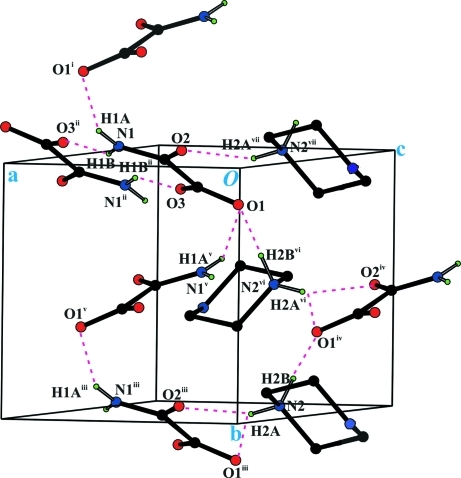
N—H···O hydrogen bonds (dotted lines) in the title compound.[Symmetry code: (i) -x+1, y-1/2, -z+1/2 ; (ii) -x+1, -y, -z; (iii) x, y+1, z; (iv)-x, y+1/2, -z+1/2 (v) -x+1, y+1/2, -z+1/2; (vi) -x, y-1/2, -z+1/2; (vii) x, y-1, z.]

## supplementary crystallographic information

### Comment

Piperazines are among the most important building blocks in today's drug discovery. The piperazine nucleus is capable of binding to multiple receptors with high affinity and therefore piperazine has been classified as a privileged structure (Dinsmore *et al.*, 2002). They are found in biologically active compounds across a number of different therapeutic areas (Berkheij *et al.*,  2005) such as antifungal, antibacterial, antimalarial, antipsychotic, antidepressant and antitumour activity against colon, prostate, breast, lung and leukemia tumors (Humle & Cherrier, 1999). Also Piperazine derivatives are widely used as buffers (Good *et al.*, 1966), and can act as complexing reagents with metal ions (Bharathi *et al.*, 2006). Encouraged by the above information, we report the crystal structure of the title compound, piperazinium bis (dioxamate) (I) (Fig. 1).

In the crystal structure of (I), the piperazinium dication lies on a centre of inversion and adopts a typical chair conformation. The bond lengths in (I) are normal and comparable with the corresponding values observed in the related structure (Wilkinson & Harrison, 2007). The dihedral angle between the piperazinium dication and oxamate anion is 9.54 (3)°. The crystal structure (Fig. 2) is stabilized by cation–to–anion N—H···O hydrogen bonds between the N—H atoms of the piperazinium ring and the O atoms of the oxamate (Fig. 2 and Table 1; symmetry code as in Fig. 2). The crystal packing is further stabilized by anion–to–anion N—H···O hydrogen bonds between the N—H atoms and the O atoms from the neighbouring oxamate anions (Fig. 2 and Table 1; symmetry code as in Fig. 2). Thus, the symmetry–related molecules are cross linked by these hydrogen bonds to generate a three–dimensional network.

### Experimental

Piperazinium bis(dioxamate) was prepared by adding aqueous solution (15ml) of piperazine (0.194g; 0.001mol) to the solution (15ml) of oxamic acid (0.089g; 0.001mol). The resulting clear solution was concentrated over water-bath to half the volume and kept for crystallization at room temperature. The transparent single crystals suitable for x-ray diffraction obtained after two days were filtered off, washed with ethanol and air dried.

### Refinement

H atoms were positioned geometrically and allowed to ride on their parent atoms, with N—H = 0.86–0.90 Å and C—H = 0.97 Å with *U*_iso_(H)= 1.2*U*_eq_.

### Crystal data

**Table:**

C_4_H_12_N_2_^2+^·2C_2_H_2_NO_3_^−^	*F*(000) = 280
*M**_r_* = 264.25	*D*_x_ = 1.555 Mg m^−^^3^
Monoclinic, *P*2_1_/*c*	Mo *K*α radiation, λ = 0.71073 Å
Hall symbol: -P 2ybc	Cell parameters from 2743 reflections
*a* = 6.4323 (4) Å	θ = 3.1–36.3°
*b* = 6.7681 (4) Å	µ = 0.13 mm^−^^1^
*c* = 13.0032 (7) Å	*T* = 293 K
β = 94.488 (2)°	Block, colourless
*V* = 564.35 (6) Å^3^	0.24 × 0.22 × 0.16 mm
*Z* = 2	

### Data collection

**Table:**

Bruker APEXII CCD diffractometer	2606 independent reflections
Radiation source: fine-focus sealed tube	2197 reflections with *I* > 2σ(*I*)
graphite	*R*_int_ = 0.021
Detector resolution: 10.0 pixels mm^-1^	θ_max_ = 36.3°, θ_min_ = 3.1°
ω scans	*h* = −10→10
Absorption correction: multi-scan (*SADABS*; Sheldrick, 1996)	*k* = −11→10
*T*_min_ = 0.969, *T*_max_ = 0.979	*l* = −20→9
9313 measured reflections	

### Refinement

**Table:**

Refinement on *F*^2^	Primary atom site location: structure-invariant direct methods
Least-squares matrix: full	Secondary atom site location: difference Fourier map
*R*[*F*^2^ > 2σ(*F*^2^)] = 0.041	Hydrogen site location: difference Fourier map
*wR*(*F*^2^) = 0.119	H-atom parameters constrained
*S* = 1.09	*w* = 1/[σ^2^(*F*_o_^2^) + (0.0667*P*)^2^ + 0.0606*P*] where *P* = (*F*_o_^2^ + 2*F*_c_^2^)/3
2606 reflections	(Δ/σ)_max_ < 0.001
82 parameters	Δρ_max_ = 0.36 e Å^−^^3^
3 restraints	Δρ_min_ = −0.34 e Å^−^^3^

### Special details

**Table:**

Geometry. All esds (except the esd in the dihedral angle between two l.s. planes) are estimated using the full covariance matrix. The cell esds are taken into account individually in the estimation of esds in distances, angles and torsion angles; correlations between esds in cell parameters are only used when they are defined by crystal symmetry. An approximate (isotropic) treatment of cell esds is used for estimating esds involving l.s. planes.
Refinement. Refinement of F^2^ against ALL reflections. The weighted R-factor wR and goodness of fit S are based on F^2^, conventional R-factors R are based on F, with F set to zero for negative F^2^. The threshold expression of F^2^ > 2sigma(F^2^) is used only for calculating R-factors(gt) etc. and is not relevant to the choice of reflections for refinement. R-factors based on F^2^ are statistically about twice as large as those based on F, and R- factors based on ALL data will be even larger.

### Fractional atomic coordinates and isotropic or equivalent isotropic displacement parameters (Å^2^)

**Table:**

	*x*	*y*	*z*	*U*_iso_*/*U*_eq_	
N2	0.08666 (10)	0.95960 (10)	0.40409 (4)	0.02372 (13)	
H2A	0.1752	0.9900	0.3562	0.028*	
H2B	0.0161	0.8502	0.3830	0.028*	
C3	−0.06294 (12)	1.12524 (12)	0.41301 (5)	0.02590 (15)	
H3A	−0.1436	1.1441	0.3475	0.031*	
H3B	0.0132	1.2462	0.4297	0.031*	
C4	0.20790 (11)	0.91851 (12)	0.50401 (6)	0.02572 (15)	
H4A	0.2938	1.0320	0.5240	0.031*	
H4B	0.2992	0.8064	0.4962	0.031*	
O1	0.13426 (8)	0.15896 (9)	0.19612 (4)	0.02683 (13)	
O2	0.44704 (10)	−0.03929 (12)	0.30376 (4)	0.03431 (16)	
O3	0.28309 (12)	0.09059 (12)	0.05073 (4)	0.03678 (17)	
N1	0.61883 (11)	−0.06196 (13)	0.15969 (5)	0.03142 (17)	
H1A	0.7247	−0.1186	0.1917	0.038*	
H1B	0.6182	−0.0387	0.0946	0.038*	
C1	0.27471 (10)	0.08954 (10)	0.14582 (4)	0.02114 (13)	
C2	0.45721 (11)	−0.01070 (11)	0.21043 (5)	0.02200 (14)	

### Atomic displacement parameters (Å^2^)

**Table:**

	*U*^11^	*U*^22^	*U*^33^	*U*^12^	*U*^13^	*U*^23^
N2	0.0244 (3)	0.0285 (3)	0.0190 (2)	−0.0053 (2)	0.00670 (19)	−0.0034 (2)
C3	0.0285 (3)	0.0280 (3)	0.0214 (3)	−0.0015 (3)	0.0027 (2)	0.0021 (2)
C4	0.0208 (3)	0.0312 (4)	0.0254 (3)	0.0001 (2)	0.0034 (2)	−0.0022 (2)
O1	0.0230 (2)	0.0337 (3)	0.0242 (2)	0.0066 (2)	0.00426 (18)	0.00091 (19)
O2	0.0304 (3)	0.0553 (4)	0.0179 (2)	0.0129 (3)	0.0059 (2)	0.0084 (2)
O3	0.0432 (4)	0.0502 (4)	0.0168 (2)	0.0188 (3)	0.0017 (2)	0.0017 (2)
N1	0.0291 (3)	0.0460 (4)	0.0200 (2)	0.0156 (3)	0.0067 (2)	0.0051 (2)
C1	0.0232 (3)	0.0222 (3)	0.0181 (2)	0.0024 (2)	0.0014 (2)	0.0006 (2)
C2	0.0225 (3)	0.0261 (3)	0.0178 (3)	0.0038 (2)	0.0039 (2)	0.0019 (2)

### Geometric parameters (Å, °)

**Table:**

N2—C3	1.4879 (11)	C4—H4B	0.9700
N2—C4	1.4883 (10)	O1—C1	1.2478 (5)
N2—H2A	0.9000	O2—C2	1.2357 (8)
N2—H2B	0.9000	O3—C1	1.2419 (5)
C3—C4^i^	1.5095 (10)	N1—C2	1.3205 (9)
C3—H3A	0.9700	N1—H1A	0.8600
C3—H3B	0.9700	N1—H1B	0.8600
C4—C3^i^	1.5095 (10)	C1—O3	1.2419 (5)
C4—H4A	0.9700	C1—C2	1.5459 (10)
			
C3—N2—C4	111.75 (6)	N2—C4—H4B	109.6
C3—N2—H2A	109.3	C3^i^—C4—H4B	109.6
C4—N2—H2A	109.3	H4A—C4—H4B	108.1
C3—N2—H2B	109.3	C2—N1—H1A	120.0
C4—N2—H2B	109.3	C2—N1—H1B	120.0
H2A—N2—H2B	107.9	H1A—N1—H1B	120.0
N2—C3—C4^i^	110.33 (6)	O3—C1—O1	127.54 (7)
N2—C3—H3A	109.6	O3—C1—O1	127.54 (7)
C4^i^—C3—H3A	109.6	O3—C1—C2	116.96 (6)
N2—C3—H3B	109.6	O3—C1—C2	116.96 (6)
C4^i^—C3—H3B	109.6	O1—C1—C2	115.50 (5)
H3A—C3—H3B	108.1	O2—C2—N1	123.63 (7)
N2—C4—C3^i^	110.48 (6)	O2—C2—C1	120.41 (6)
N2—C4—H4A	109.6	N1—C2—C1	115.96 (5)
C3^i^—C4—H4A	109.6		
			
C4—N2—C3—C4^i^	−56.67 (9)	O3—C1—C2—O2	−170.89 (8)
C3—N2—C4—C3^i^	56.75 (9)	O1—C1—C2—O2	8.52 (11)
O3—O3—C1—O1	0.00 (4)	O3—C1—C2—N1	8.95 (11)
O3—O3—C1—C2	0.00 (4)	O3—C1—C2—N1	8.95 (11)
O3—C1—C2—O2	−170.89 (8)	O1—C1—C2—N1	−171.64 (7)

Symmetry codes: (i) −*x*, −*y*+2, −*z*+1.

### Hydrogen-bond geometry (Å, °)

**Table:**

*D*—H···*A*	*D*—H	H···*A*	*D*···*A*	*D*—H···*A*
N1—H1A···O1^ii^	0.86	2.24	3.0232 (9)	152
N1—H1B···O3^iii^	0.86	2.07	2.8622 (8)	153
N2—H2A···O1^iv^	0.90	2.37	3.0589 (8)	133
N2—H2A···O2^iv^	0.90	1.94	2.7475 (9)	149
N2—H2B···O1^v^	0.90	1.87	2.7509 (9)	164

Symmetry codes: (ii) −*x*+1, *y*−1/2, −*z*+1/2; (iii) −*x*+1, −*y*, −*z*; (iv) *x*, *y*+1, *z*; (v) −*x*, *y*+1/2, −*z*+1/2.
